# Electricity Recovery from Municipal Sewage Wastewater Using a Hydrogel Complex Composed of Microbially Reduced Graphene Oxide and Sludge

**DOI:** 10.3390/ma9090742

**Published:** 2016-08-31

**Authors:** Naoko Yoshida, Yasushi Miyata, Ai Mugita, Kazuki Iida

**Affiliations:** 1 Department of Civil Engineering, Nagoya Institute of Technology, Nagoya 466-8555, Japan; 2 Nagoya Municipal Industrial Research Institute, 3-4-41, Rokuban, Atsuta-ku, Nagoya 456-0058, Japan; miyata@nmiri.city.nagoya.jp; 3 Nippon Koei Co., Ltd., 1-14-6 Kudankita, Chiyoda-ku, Tokyo 102-8539, Japan; a7138@n-koei.co.jp (A.M.); a4609@n-koei.co.jp (K.I.)

**Keywords:** graphene oxide, municipal sewage wastewater, microbial fuel cell, *Geobacter*, *Geothrix*

## Abstract

Graphene oxide (GO) has recently been shown to be an excellent anode substrate for exoelectrogens. This study demonstrates the applicability of GO in recovering electricity from sewage wastewater. Anaerobic incubation of sludge with GO formed a hydrogel complex that embeds microbial cells via π-π stacking of microbially reduced GO. The rGO complex was electrically conductive (23 mS·cm^−1^) and immediately produced electricity in sewage wastewater under polarization at +200 mV vs. Ag/AgCl. Higher and more stable production of electricity was observed with rGO complexes (179–310 μA·cm^−3^) than with graphite felt (GF; 79–95 μA·cm^−3^). Electrochemical analyses revealed that this finding was attributable to the greater capacitance and smaller internal resistance of the rGO complex. Microbial community analysis showed abundances of *Geobacter* species in both rGO and GF complexes, whereas more diverse candidate exoelectrogens in the Desulfarculaceae family and *Geothrix* genus were particularly prominent in the rGO complex.

## 1. Introduction

The application of bioelectrochemical systems (BESs), such as microbial fuel cells (MFCs) and microbial electrolysis cells (MECs), in wastewater treatment has received substantial attention because these approaches offer several advantages in recovering electricity from organic matter, such as the reduction of aeration and the production of less excess sludge [1]. Conventional activated-sludge treatment requires aeration for a constant oxygen supply and an electric sink that consumes 0.3 kWh·m^−3^ of electric power, which is approximately two times more than the electric energy used in the entire system [2]. In BESs, microbial degradation is performed without aeration in an anodic chamber where microorganisms degrade organic matter and transfer electrons to the anode. Electrons recovered in the anode are later utilized to reduce oxygen or for the synthesis of valuable materials at the cathode. MFCs can typically generate electricity at 2–3 W·m^−2^ of the projected area of the cathode [1], although the power density varies between MFCs with different configurations and wastewaters. The maximum power density reported with domestic wastewater and an MFC was 12 W·m^−3^ [3], which is equivalent to 0.07 kWh·m^−3^. The energy recovery is low, considering that domestic wastewater contains ~2 kW·h^−1^·m^−3^ [4]. This indicates a need for technical improvements that can increase the recovery of energy using MFCs.

The anode is the critical factor that affects energy recovery in BESs. To obtain better performance, the anode must show good affinity for microbes and have a large surface area to allow the adhesion of a large number of microbial cells [5,6]. Recently, the 3D structures of the anode using various materials such as layered corrugated carbon, graphene, and steel fiber were found to greatly facilitate energy recovery because they can achieve efficient proton/electron transfer in the anode-microbial biofilm complex [7,8,9]. Graphene oxide (GO), the oxidized form of graphene, has been also reported to facilitate electricity production in several MFCs [10].

GO itself is not electrically conductive due to its disrupted sp^2^ bonding network and it cannot facilitate electron transfer [11]. However, the reduced form of GO (rGO) is electrically conductive because the π-network is restored via reduction [11] and can be produced by bacteria [12,13]. Furthermore, GO has a relatively large surface area (similar to graphene) [14] and the colloidal properties of GO and bacteria enable efficient aggregation in aqueous solutions [15]. In addition, GO can serve as an electron acceptor for the selective growth of exoelectrogens [16,17] and self-aggregate into a 3D-conductive hydrogel that embeds exoelectrogens via microbial reduction.

The microbial reduction of GO was first demonstrated using *Shewanella* species [12,13] and then later with *Escherichia coli* [18] and natural microcosms [19,20]. The addition of GO into those cultures or microcosms with soil particles led to the production of rGO as a floc precipitate [19,20,21]. Recently, we showed that, with acetate as the sole energy and carbon source, the growth of exoelectrogenic bacteria such as *Geobacter* [16] and *Desulfovibrio* species [17] is dependent on GO reduction, and these bacteria can produce a hydrogel complex with rGO. The rGO-*Geobacter* complex showed considerably more stable energy production than did graphite felt (GF), which can be attributed to better biofilm growth, greater electric double-layer capacitance and much smaller charge-transfer resistance [16]. However, all of these experiments were performed in highly enriched cultures using synthetic minimum medium containing acetate as the sole energy and carbon source. Specifically, the enriched cultures mainly consisted of several species, i.e., bacteria of the *Geobacter*, *Azospira*, and *Desulfovibrio* genera comprising >90% of the bacteria detected. Under such simple conditions, most electrons of the consumed acetate molecules were recovered as electricity via the rGO complex (≥90% Coulomb efficiency) [16]. However, sewage wastewater contains various organic and inorganic compounds and generally consists of thousands of different species of microorganisms [22]. In such complex environments, the recovery of electrons on GO/rGO can be highly competitive with other metabolisms (i.e., other respiration and methanogenesis pathways). Therefore, it is not clear whether the self-aggregation of hydrogel anodes and better performance in terms of electricity recovery can be reproduced in sewage wastewater.

In this study, we evaluated the applicability of GO in enriching for exoelectrogens and recovering electricity from sewage wastewater. Specifically, we attempted to directly prepare a hydrogel anode by mixing GO and anaerobic sludge without an enrichment process for future practical applications. The mixture of GO and sludge successfully self-aggregated into a conductive hydrogel complex (the rGO complex) that embeds sludge via partial π-π stacking in rGO. Polyphasic characterization showed better performance of the rGO complex than the GF-sludge complex (GF complex) in terms of electricity recovery and biofilm growth, which involved diverse exoelectrogens such as members of the *Geobacter* and *Geothrix* genera and the Desulfarculaceae family.

## 2. Results and Discussion

### 2.1. GO Reduction in Anaerobic Sludge

**Figure 1:**
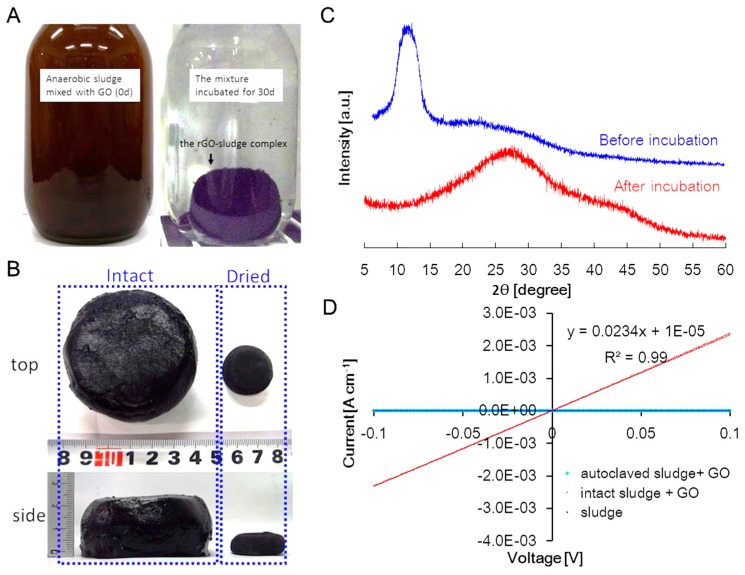
Graphene oxide (GO) and anaerobic sludge mixture changes, and the formation of a hydrogel complex of reduced GO and sludge. (**A**) GO reduction in the anaerobic sludge; (**B**) Changes in volume of the rGO complex before and after drying; (**C**) XRD patterns of GO and the rGO complex before and after incubation; (**D**) Conductivity of the autoclaved sludge with GO, the intact sludge with GO, and the intact sludge without GO.

The microbial reduction of GO using anaerobic sludge was conducted by the anaerobic incubation of sludge mixed with GO. Figure 1A shows the apparent changes in the culture before and after incubation. At day 0, the GO in the mixture was well dispersed in the entire culture, which was brown in color, and the sludge settled down at the bottom of a vial. During the 30 days of incubation, the mixture changed into a black semi-solid complex of cylindrical shape.

Figure 1B shows a black semi-solid complex taken from a vial. The black color was typical of rGO [11], suggesting that microbial reduction of GO occurred. The volume of the semi-solid complex was reduced by 96% and the weight was reduced by 94% after air-drying. These findings indicated that the complex was a porous 3D hydrogel material containing a substantial degree of water (96%, *v*/*v*) [23].

Figure 1C shows the X-ray diffraction (XRD) patterns of the hydrogel complex. The original mixture of GO and sludge showed a broad peak at 2*θ* = 10.4° corresponding to a d-space of 8.5 Å, which was similar to a typical broad peak for GO (i.e., 2*θ* = 10.27°) [24]. After anaerobic incubation, the GO peak disappeared and another broad peak appeared at 2*θ* = 26.5°, which corresponded to a d-space of 3.4 Å. The new peak was similar to that of graphite (2*θ* = 26.6°) [25]. Decreased d-spacing is due to the removal of oxygen and water from the interlayers by reduction [11]. Our results were in good agreement with the XRD spectra of rGO after GO reduction by other microorganisms [26,27] and hydrothermally reduced GO [28]. These results clearly indicated that complexed GO was reduced and changed into rGO comprised of stacked multilayers. The π-π stacking of rGO was a probable mechanism of the self-aggregation of the complex of rGO and sludge.

The electrical conductivity of the hydrogel complex was determined by linear-sweep voltammetry using four sensing probes (Figure 1D). The hydrogel complex showed a conductivity of 23 mS·cm^−1^, although no significant electric conductivity was observed in intact sludge or in the mixture of autoclaved sludge and GO. The increase in conductivity after anaerobic incubation agreed with the XRD data showing the reduction of GO to conductive rGO.

These changes in the color, XRD pattern, and electrical conductivity of the sludge and GO mixture indicated that the produced hydrogel complex was the complex of conductive rGO and sludge via the π-π stacking of rGO. The formed complex is defined as the rGO complex hereafter.

### 2.2. Electrochemical Cultivation of the rGO-Sludge Complex

**Figure 2:**
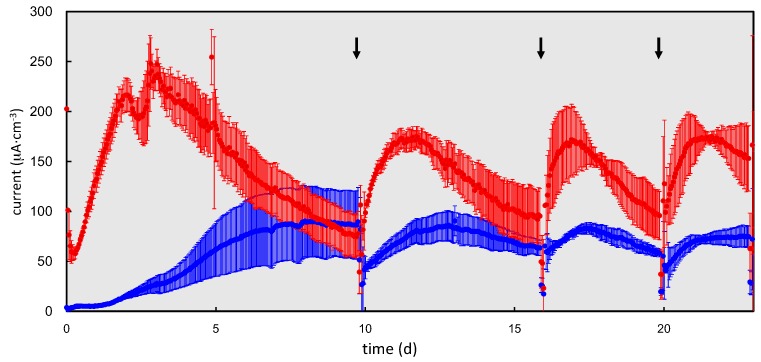
The production of electricity from sewage wastewater using the rGO and GF complexes. The data shown represent the average values of three independent experiments performed in parallel. The arrows indicate when the wastewater in the bottles was replaced. The error bars are standard errors from three independent experiments.

To ensure that the rGO complex can produce electricity from organic matter in sewage wastewater, the rGO complex was polarized at +200 mV (vs. Ag/AgCl) in fresh sewage wastewater, and the GF complex was polarized in parallel for comparison purposes. Figure 2 shows the production of electricity using both complexes. The rGO complexes produced electricity up to 250 μA·cm^−3^ within two days, whereas the electricity produced by the GF complexes gradually increased to 100 μA·cm^−3^ after 10 days. The electricity produced by the rGO complexes gradually decreased after peak production was reached on days 2–3. The changes of the chemical oxygen demand (COD_Cr_) values that occurred in both cultures during electric cultivation are shown in Supplemental Figure S2. The initial COD_Cr_ concentrations differed between the two cultures, although sewage wastewater having a density of 220 mg·L^−1^ was introduced into the two cultures. The densities of the rGO and GF cultures were 150 ± 5.0 mg·L^−1^ and 250 ± 7.4 mg·L^−1^, respectively. The higher initial COD_Cr_ in the GF culture was probably due to the elution of organic matter in the culture, which was injected with sludge. At day 10, the level of COD_Cr_ in the culture with the rGO complex was under the detection limit, whereas it remained at 220 ± 36 mg·L^−1^ in cultures with the GF complex. These data suggested that the observed decrease of electricity in the rGO complexes, which occurred earlier than that in the GF complex, was most likely caused by a decreased availability of organic matter. Electricity production in cultures containing the rGO complex was recovered by replacing the sewage wastewater on day 10. This trend was repeatedly observed for both complexes. The rGO complex tended to produce more electricity overall than the GF complex did. In the rGO complex, the peak electricity in the second to fourth cycles (10–23 days) was 180–210 μA·cm^−3^, which was lower than that produced in the first cycle. It is possible that the decrease in peak electricity production was due to a decreased number of available surface pores caused by the biofilm grown in the first cycle.

One sample from each triplicate culture was continuously incubated over 100 days and showed differences in electricity production (Supplementary Materials Figure S3), while the remaining two cultures were used for other experiments. Long-term polarizations showed a gradual decrease, but stable electricity production (peak production: 63–160 μA·cm^−3^) in the rGO-sludge complex over a 200 day incubation. However, the GF complex showed decreased electricity production (<μA·cm^−3^) after the fifth replacement at 50 days and did not recover following the replacement of wastewater.

**Figure 3:**
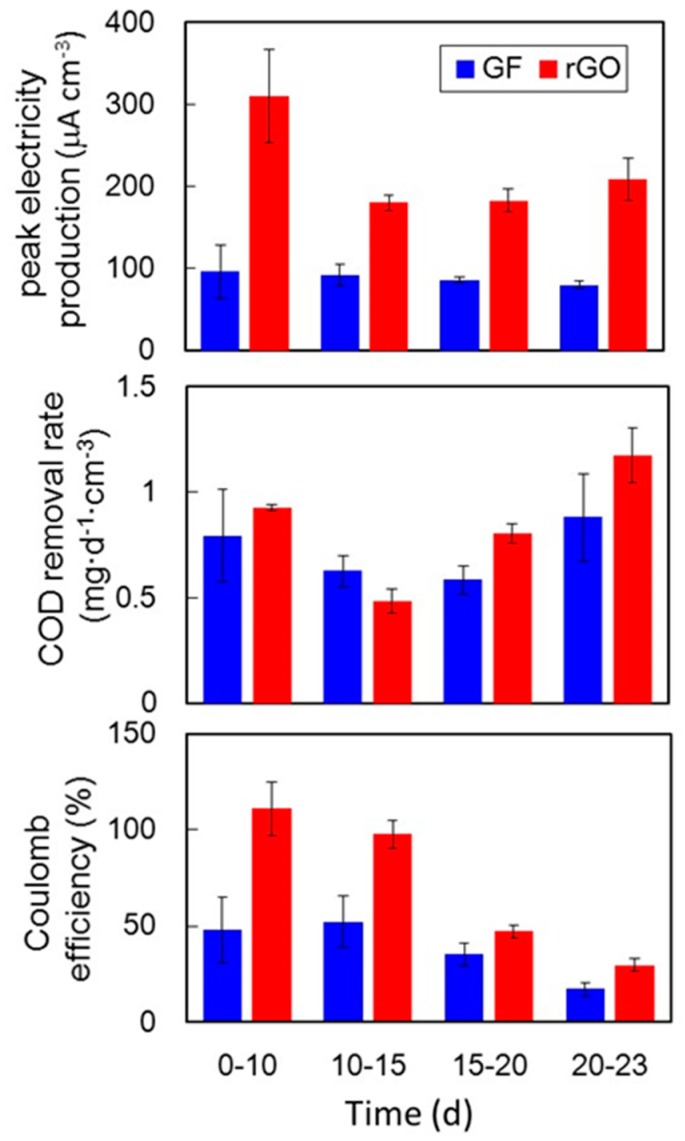
COD_Cr_ removal and energy recovery efficiency in the rGO and GF complexes. The data shown represent the average values of three independent experiments, performed in parallel. The error bars are standard errors of the three determinations

The performances of the two investigated culture methods in four-batch feeding are summarized in Figure 3. Peak electricity production in each batch feeding ranged from 180 to 310 μA·cm^−3^ for the rGO complexes, which was approximately two- to three-fold higher than production by the GF complexes (Figure 3).

The COD removal rates in the two cultures were 0.48–1.2 mg·day^−1^·cm^−3^ and did not differ significantly (Figure 3). Based on the electricity recovery rates and COD removal rates, coulombic efficiencies (CEs) were 30%–110% for the rGO complex, which were significantly higher than those for the GF complex (17%–52%) overall, although this difference at day 15–20 was small and not significant (Figure 3). Higher CEs using the rGO complex were potentially found because the rGO complex recovered energy better than the GF complex did.

### 2.3. Biomass Analysis of the rGO and GF Complexes

Both complexes were prepared using 2000 mg anaerobic sludge. The cell density of the rGO complex was (2.4 ± 0.17) × 10^8^ cells cm^−3^, which was approximately six-fold higher than that in liquid culture (Table 1). This finding suggested that the cells in anaerobic sludge were enriched in the rGO complex. Of the total biomass in the culture, 13% ± 2.8% was present in the complex (approximately 18 cm^3^), whereas 86% was planktonic in the 900 mL liquid culture. The cell density in the GF complex was (4.0 ± 1.0) × 10^8^ cells cm^−3^, which accounted for 20% ± 4.9% of the total biomass. The differences of the two values compared to those found with the rGO complex were not statistically significant (*p* > 0.05).

**Figure 4:**
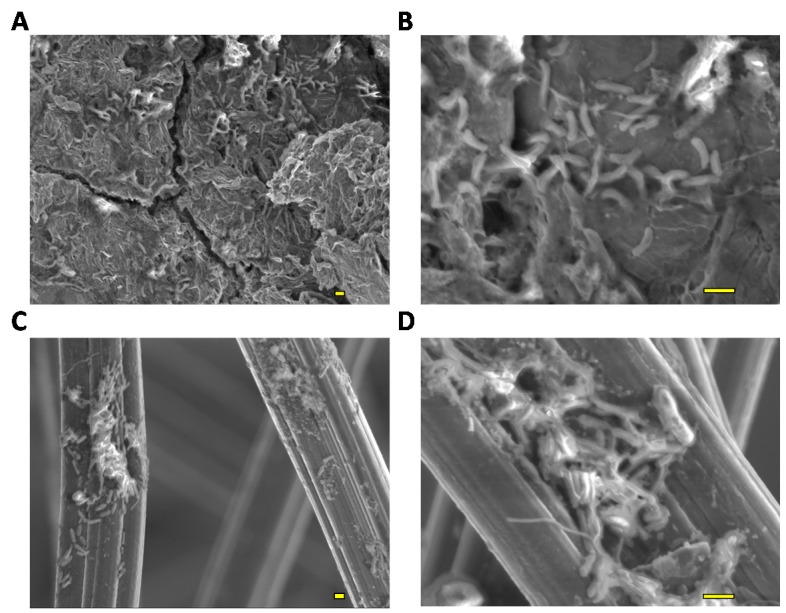
SEM images of biofilm growth on the rGO (**A**,**B**) and GF complexes (**C**,**D**). Scale bars on images indicate 1 μm.

After 23 days of polarization, the rGO complex showed an increased number of cells compared with the GF complex. A cell density of (8.4 ± 1.4) × 10^8^ cells cm^−3^ was observed in the rGO complex, which was 1.8-fold higher than that in the GF complex. This finding indicated that rGO promoted better biofilm growth than GF did. The biomass in the rGO complex accounted for 38% ± 12% of the total biomass of the culture, whereas the biomass in the GF complex was limited to 14% ± 1.7% of the total biomass. The higher proportion of the biofilm cells in the rGO complex was attributed to the enhancement of the biofilm formation by rGO and the increase of planktonic cells in the GF culture. SEM observations of both complexes supported the cell-counting results and showed that biofilm growth occurred more with rGO complexes than with GF complexes (Figure 4).

The total biomass in cultures after polarization was lower with rGO (Table 1) than with GF, showing greater growth of planktonic cells in liquid phase culture using GF. Compared with the results following COD_Cr_ removal and biomass growth during a 23 days incubation, the assimilation rate was lower in cultures using rGO complexes ((1.1 ± 3.3) × 10^7^ cells mg-COD_Cr_^−1^) than observed when using GF complexes ((8.8 ± 1.3) × 10^7^ cells mg-COD_Cr_^−1^).

### 2.4. Electrochemical Comparison of the rGO and GF Complexes

**Figure 5:**
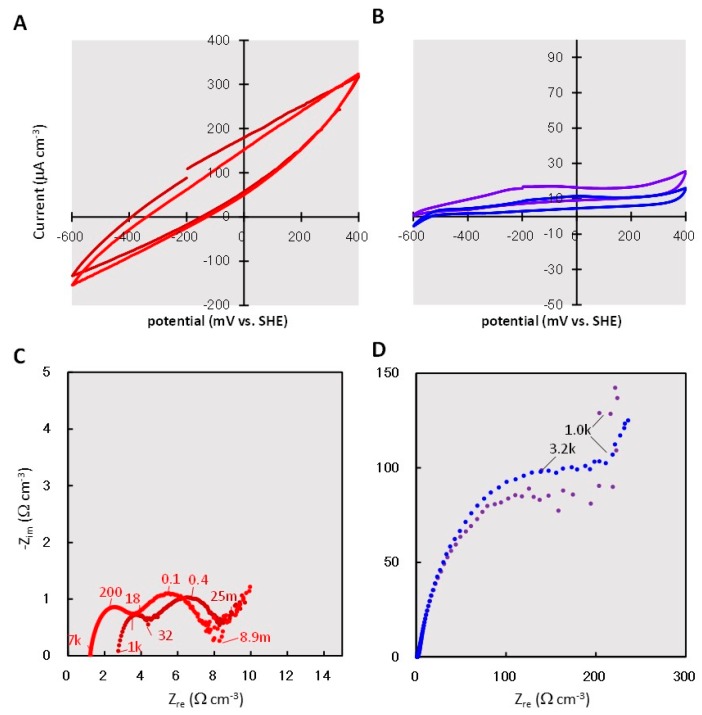
Cyclic voltammetry (CV) and electrochemical impedance spectroscopy (EIS) data for the rGO and GF complexes after polarization. A and B show CV data for the rGO complex (**A**) and GF complex (**B**). C and D are EIS data for the rGO complex (**C**) and the GF complex (**D**). Data for each are presented from two independent cultures, maintained in parallel. The numbers shown in graphs C and D are frequencies: f (Hz) = ω·2π^−1^.

Figure 5 shows the CV curves of the rGO (Figure 5A) and GF (Figure 5B) complexes. The rGO complex showed a higher catalytic current than the GF complex did. For example, the catalytic current of the rGO complex was 320 μA·cm^−3^ at 400 mV vs. Ag/AgCl, whereas that of the GF complex was below 30 μA·cm^−3^ at 400 mV vs. Ag/AgCl. The voltammograms for the rGO complex showed symmetric discharges with large closed areas, indicating that the rGO complexes had a larger electric double-layer capacitance than the GF complexes did, due to the larger surface area of rGO.

Nyquist plots were obtained for the rGO (Figure 5C) and GF (Figure 5D) complexes. The charge-transfer resistances (*R*_ct_), represented as the diameter of the semicircles, were estimated to be <10 Ω·cm^−3^ in the rGO complexes. In contrast, *R*_ct_ in the GF complexes was >200 Ω·cm^−3^. In ideal electrochemical kinetic reactions, the capacitance (C) is inversely proportional to *R*_ct_, and the angular frequency (ω_max_) shows the top of the semicircle (ω_max_*CR*_ct_ = 1). Therefore, the capacitance in the rGO complex was estimated to be much higher than that in the GF complex.

### 2.5. Comparison of Microbial Communities in the rGO and GF Complexes

The microbial compositions of the rGO and GF complexes were analyzed by high-throughput sequencing of 16S rRNA gene amplicons. The numbers of reads for the rGO and GF complexes were 54,065 and 49,811, respectively. An operation taxonomic unit (OTU) was defined as a phylogenetic group having ≥97% sequence similarity, and 1535 and 1993 OTUs were found for the rGO and GF complexes, respectively.

**Figure 6:**
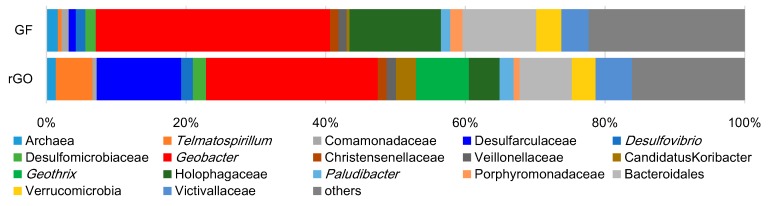
Microbial community structures in the rGO and GF complexes. The data were obtained from single experiments using complexes polarized for 30 days.

Despite distinct differences in the electrochemical properties, both complexes had similar microbial compositions with predominant *Geobacter* species (Figure 6). *Geobacter* species are well-known exoelectrogenic bacteria, and the high percentage of these bacteria has been crucial for enhanced electricity production. However, the rGO complex, which produced more electricity, had a lower proportion (25%) of *Geobacter* species than the GF complex (34%) did. When the total biomass (Table 1) was considered, the populations of the *Geobacter* species were similar in the rGO complex (2.1 × 10^8^ cells·cm^−3^) and the GF complex (1.6 × 10^8^ cells cm^−3^).

Notable differences in the microbial compositions were observed for the Desulfarculaceae family and the *Geothrix* and *Telmatospirillum* genera. The phylotypes belonging to the Desulfarculaceae family comprised 12% of the prokaryotes in the rGO complex, but only 5% of those in the GF complex. The *Geothrix* and *Telmatospirillum* genera comprised 7.6% and 5.3% of the rGO complex, respectively, but less than 1% each of the GF complex.

## 3. Discussion

In this study, we focused on the application of GO in a self-aggregated anode complex to recover electricity from municipal sewage wastewater. The results indicated that anaerobic cultivation of an anaerobic sludge with GO could produce a conductive hydrogel complex within 30 days. Various forms of rGO and cell mixtures were analyzed in previous studies. *Shewanella* species were shown to produce floccular aggregates of rGO and cells following a three-day incubation with 0.2 g·L^−1^ GO and lactate [26]. In addition, *E. coli* transformed 0.5 g·L^−1^ GO to water-dispersed rGO after three days of anaerobic incubation with stirring [18]. In our previous studies, we observed the formation of rGO hydrogel complexes upon static cultivation of microbial cells with 0.67 g·L^−1^ GO and acetate, although the time required for solidification varied between five and seven days for the enrichment culture [17] and up to one month for a pure *Geobacter* culture [17]. The electrical conductivity of the rGO complex was similar to that in an enrichment culture of *Desulfovibrio* species (25 mS·cm^−1^) [17] and higher than in a pure culture of *Geobacter* sp. R4 (16 mS·cm^−1^) [17]. The size and electrical conductivity of rGO may depend on π-π stacking structures in the rGO complex.

The rGO complex showed higher production of electricity from municipal sewage wastewater than the GF complex did, which was likely due to its larger electric double-layer capacitance, considerably lower charge-transfer resistance, and improved biofilm growth. This is consistent with the results obtained using a pure culture of *Geobacter* sp. R4 [16]. These results indicate that GO is broadly applicable as an anodic material for the enhancement of electron recovery from microbial cells in both pure culture and complexed community.

In pure cultures of exoelectrogenic bacteria, the biomass yield generally correlates with the production of electricity. However, the total biomass in the culture using the rGO complex was less than that of the GF complex, despite the higher electricity production. Greater total biomass growth on GF was also observed in our previous study using *Geobacter* sp. R4, which was attributed to the high growth rate of planktonic cells [16]. It has been reported that planktonic cells are less involved in the production of electricity than cells in biofilm [29,30]. Hence, the change in the microbial physiological state (i.e., planktonic or biofilm) also contributed to differences in the electron recovery efficiency between the two cultures. The enhancement of bacterial attachment on rGO is possibly attributable to unsaturated and oxidized carbon remaining in the rGO after microbial reduction. Such organic molecules make the surface hydrophilic and allow the instantaneous adhesion of cells to the anode surface and the growth of exoelectrogens [31,32].

The Nyquist plot in Figure 5C shows multiple semicircles for the rGO complex, indicating the existence of multiple bioelectrochemical reactions with different charge-transfer rates in the rGO complex. In agreement, potentially diverse exoelectrogens were observed for the rGO complex in the phylogenetic identification of dominant prokaryotes. Plausible exoelectrogen candidates in the rGO complex are bacteria of the Desulfarculaceae family and the *Geobacter* and *Geothrix* genera. It was previously demonstrated that *Geothrix* species can produce electricity [33] and are frequently detected in MFCs [34,35], suggesting the involvement of these bacteria, together with *Geobacter* species, in the production of electricity from sewage wastewater. The Desulfarculaceae family contains a single isolated sulfate-reducer strain, *Desulfarculus baarsii* strain 2st14^T^ [36], and has never been detected in MFCs. However, the 2st14^T^ strain can reduce uranium (IV) [37] and can oxidize higher fatty acids completely to CO_2_ [36] via the Wood-Ljungdahl pathway [38]. Therefore, the high detection frequency (12%) of the Desulfarculaceae family in the rGO culture suggests their involvement in extracellular electrons transferring coupled with the mineralization of higher fatty acids. Members of the *Telmatospirillum* genus were detected at a 5.3% frequency in the rGO culture. The *Telmatospirillum* genus includes only a single species, *T. siberiense*. All strains of *T. siberiense* grow by aerobic respiration, while the bacteria can grow via fermentation only under anoxic conditions [39] and have never been assayed for electricity production. *T. siberiense* has been detected in some MFCs, although the detection frequency was limited to <2% [40,41,42]. It seems probable that bacteria of the *Telmatospirillum* genus in the rGO culture grow via fermentation and that their higher proportion is potentially attributable to their syntrophic growth with other anaerobic bacteria [43]. The rGO complex formation potentially enabled the involvement of various bacteria in electricity production, either directly or indirectly. The presence of multiple exoelectrogens potentially enhances the production of electricity coupled with the oxidization of different hydrocarbons. Plausible factors contributing to the diversity of exoelectrogens in the rGO complex include the non-uniform chemical structure of rGO, which facilitates cell adhesion [31,32], and myriad sites having locally different potentials inside the rGO complex [44].

## 4. Materials and Methods

### 4.1. Preparation of rGO and GF Complexes with Anaerobic Sludge

Powdered GO was purchased from Royal Elite New Energy Science & Technology Co., Ltd. (Shanghai, China) and dispersed into MilliQ water, as described previously [17]. To investigate GO reduction by anaerobic sludge and the self-aggregation of rGO and anaerobic sludge into hydrogel complexes, 0.67 g GO from a 10 g·L^−1^ GO stock solution was added to 1 L of anaerobic sludge wastewater suspension (approximately 2000 mg·L^−1^ of mixed liquor suspended solids), in a 2 L anaerobic medium bottle. Afterward, the mixture was transferred to screw-capped glass bottles (0.93 L capacity; size, 90 mm diameter and 175 mm height). Extra headspace in the bottles was removed by filling the bottles with the mixture, and then the bottles were closed. The glass bottles were incubated statically at 28 °C, without any physical manipulations. After a one-month incubation, the formed rGO complex was dehydrated by manual compression and reduced in size to a 30 mm diameter. Then, the rGO complex was used as the anode for electrochemical cultivation. A schematic representation of the experiments performed in this study is shown in Supplemental Figure S1.

For comparison purposes, GF (30 mm diameter and 20 mm thickness) was used as the representative MFC anode. One liter of anaerobic sludge suspension was condensed to 10 mL by centrifugation (8000× *g*, 10 min, room temperature), and the obtained slurry was injected into cut GF to form GF complexes.

### 4.2. X-ray Photoelectron Spectroscopy (XPS) and Scanning Electron Microscopy (SEM) Analyses

The chemical states of GO and rGO were analyzed with XPS using a Versa Probe PHI-5000 (ULVAC-PHI Inc., Osaka, Japan), as described previously (Yoshida et al., 2015b). For SEM imaging, the prepared complexes were fixed with 2% glutaraldehyde and 1% osmium tetroxide, sputter-coated with gold as described previously [16], and observed by field-emission SEM (JSM-7800F; JEOL Ltd., Tokyo, Japan) operating at 1.0 kV.

### 4.3. Direct Cell Counting and Chemical Oxygen Demand (COD) Analyses

Sample cell densities were determined by directly counting cells stained with SYBR Green II under a microscope, as described previously [45]. For cell counting in anode complexes, the complexes with GF or rGO were cut into pieces, suspended in 10 mL of 10 mM phosphate-buffered saline supplemented with 1 mM EDTA (PBSE, pH 7.2), and vortexed for 1 min. The pieces of complex suspended in PBSE buffer was serially diluted and filtered using a black polycarbonate membrane filter. The cells on the filter were observed under a BX-53 phase-contrast/epifluorescence microscope equipped with a DP72 digital camera (Olympus Corporation, Tokyo, Japan) and counted using ImageJ software ver. 1.49. We measured chemical oxygen demand COD_Cr_ using a standard colorimetric wastewater method (5220 D), as described elsewhere [21].

### 4.4. Electrochemical Cultivation

In the cultivation cell, a sterilized glass bottle (0.93 L capacity; 90 mm diameter and 175 mm height) was filled with sewage wastewater including 156–199 mg COD_Cr_·L^−1^. Afterward, the rGO or GF complex was placed in a platinum cage in the bottle and connected with a platinum wire as the working electrode. An Ag/AgCl (KCl salt) electrode and a second platinum wire were used as reference and counter electrodes, respectively. The polarization was conducted by setting the working electrode potential at +200 mV versus Ag/AgCl, using a potentiostat (HA-1510; Hokuto Denko, Tokyo, Japan). During the polarization, the electrical current was recorded using a data logger (T&D Corporation, Nagano, Japan) every 60 min.

### 4.5. Electrochemical Analysis

CV and EIS analyses of the rGO and GF complexes were conducted using an electrochemical measurement system (HZ-7000; Hokuto Denko, Tokyo, Japan). CV and EIS analyses were performed using a bottle that was previously used for the electrochemical cultivation described above. CV was conducted at a scan rate of 0.2 mV·s^−1^ in the potential range from −400 to 600 mV (vs. Ag/AgCl). EIS was performed over a frequency range of 100 kHz to 0.5 MHz at 200 mV, with a 20 mV amplitude used for the applied alternating current signal. Nyquist plots were analyzed using ZSimpWin software (Princeton Applied Research, Oak Ridge, TN, USA).

### 4.6. Microbial Composition Analysis

To analyze the microbial community structure, DNA was extracted from the rGO and GF complexes after polarization for 30 days. A partial fragment of the 16S rRNA gene (approximately 150 bp) was amplified and analyzed by high-throughput sequencing using the Illumina MiSeq platform. The 16S rRNA gene amplicon was obtained using the bacterial and archaeal consensus primers, 515F (5′-GTGCCAGCMGCCGCGGTAA-3′) and 806R (5′-GGACTACHVGGGTWTCTAAT-3′). The obtained amplicons were labeled with barcode sequences in the second PCR step and pooled for subsequent paired-end sequencing. The sequence reads passed through a quality filtering using Sickle software (version 1.33) and were trimmed using Fastx Toolkit (version 0.0.13.2). The reads filtered with the chimera program usearch (version 7.0.1090_i86linux64) and analyzed using microbial community analysis software Qiime (version 1.9.0).

## 5. Conclusions

This study demonstrated the self-aggregation of sewage sludge and GO into a conductive hydrogel that embeds sludge via partial π-π stacking of microbially reduced GO, without employing an enrichment processes. The resulting rGO complex showed better electricity production with smaller charge-transfer resistance, larger capacitance, and better biofilm growth compared to the GF complex. Microbial community analysis suggested that the Desulfarculaceae family and *Geobacter* and *Geothrix* genera are involved in electricity production in the rGO complex. This simple and easily applicable process can help expand the application of GO in BESs used to treat sewage wastewater.

## Figures and Tables

**Table 1:** Biomass comparison in the rGO and GF complexes before and after polarization.

Cell Densities	Before Polarization	After 23 Days of Polarization
rGO		
complex (10^8^ cells cm^−3^)	2.4 ± 0.17	8.4 ± 1.4
complex (10^8^ cells complex^−1^)	46 ± 3.2	160 ± 27
liquid culture (10^8^ cells mL^−1^)	0.40 ± 0.86	0.33 ± 0.10
liquid culture (10^8^ cells culture^−1^)	360 ± 81	290 ± 81
total (10^8^ cells bottle^−1^)	410 ± 75	450 ± 57
complex (%)	13 ± 2.8	38 ± 12
liquid culture (%)	87 ± 2.8	62 ± 12
GF		
complex (10^8^ cells cm^−3^)	4.0 ± 1.0	4.6 ± 0.63
complex (10^8^ cells complex^−1^)	56 ± 14	87 ± 12
liquid culture (10^8^ cells mL^−1^)	0.25 ± 0.63	0.61 ± 0.10
liquid culture (10^8^ cells culture^−1^)	220 ± 55	550 ± 75
total (10^8^ cells bottle^−1^)	280 ± 69	640 ± 87
complex (%)	20 ± 4.9	14 ± 1.7
liquid culture (%)	80 ± 4.9	86 ± 0.58

## Supplementary Materials

The following are available online at www.mdpi.com/1996-1944/9/9/742/s1. Figure S1: Schematic representation of the experiments performed in this study; Figure S2: Changes of COD concentrations in the electrochemically cultivated cultures using two different complexes; Figure S3: Long-term polarization of the rGO-sludge and GF-sludge complexes in sewage wastewater.
