# Supplementary material for: Electricity Recovery from Municipal Sewage Wastewater Using a Hydrogel Complex Composed of Microbially Reduced Graphene Oxide and Sludge
Source: Materials (Basel). 2016 Aug 31;9(9):742. doi: 10.3390/ma9090742 (PMC5457117; doi:10.3390/ma9090742)
Supplement: Supplementary file 1 [file materials-09-00742-s001.pdf]

Supplementary Materials: Electricity Recovery from Municipal Sewage Wastewater Using a Hydrogel Complex Composed of Microbially Reduced Graphene Oxide and Sludge

Naoko Yoshida, Yasushi Miyata, Ai Mugita and Kazuki Iida

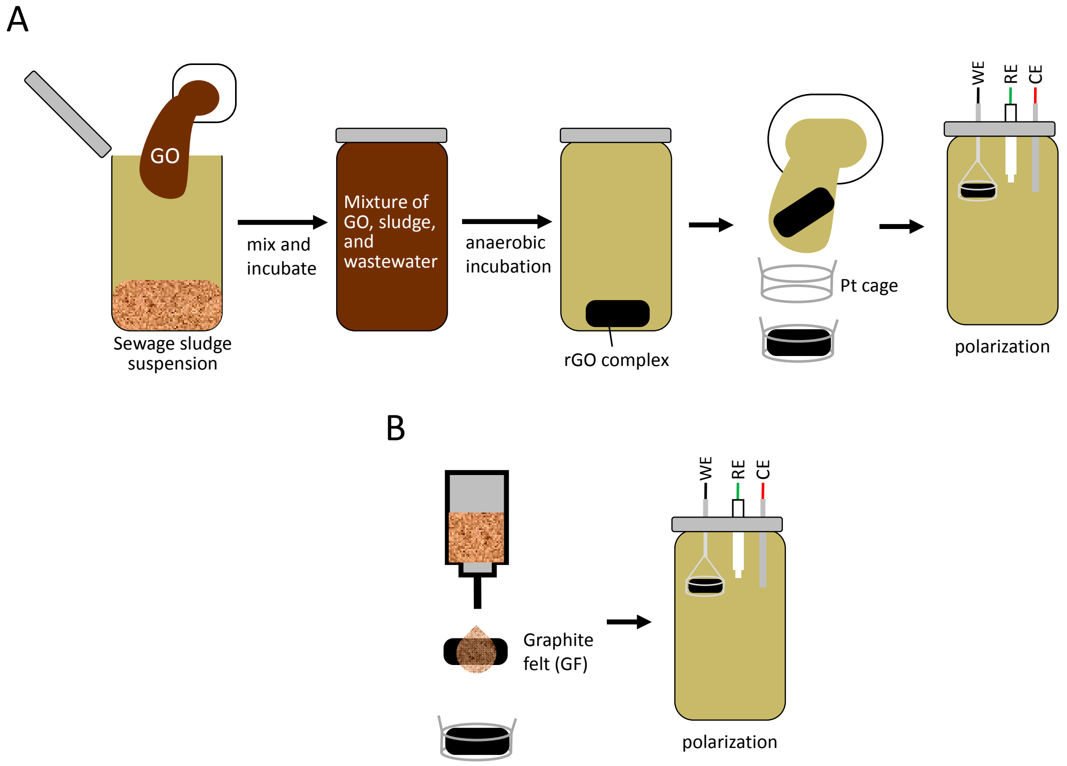

Figure S1. Schematic representation of the experiments performed in this study. The preparation and polarization procedures for the sludge-carbon complexes using graphene oxide (**A**) and graphite felt (**B**) are shown.

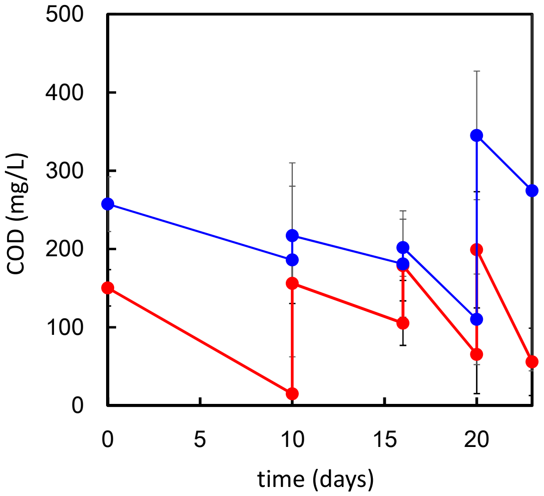

Figure S2. Changes of COD concentrations in the electrochemically cultivated cultures using two different complexes with rGO (red) and GF (blue).

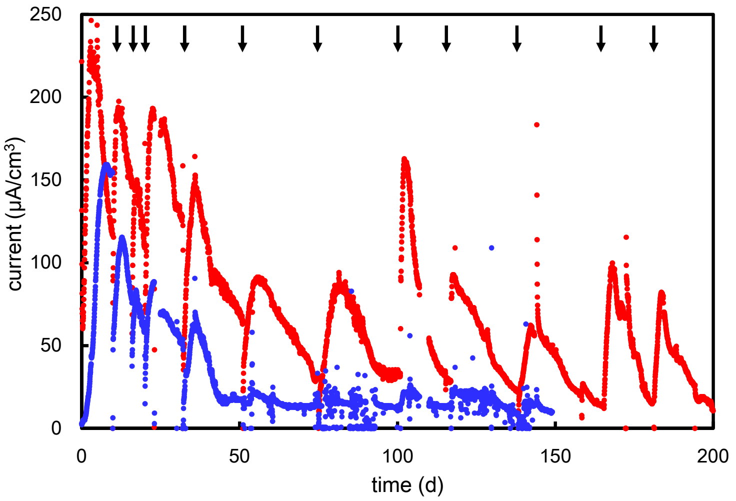

Figure S3. Long-term polarization of the rGO-sludge (red) and GF-sludge (blue) complexes in sewage wastewater. The arrows indicate the timing of wastewater replacements.
